# Investigation of Endogenous Retrovirus Sequences in the Neighborhood of Genes Up-regulated in a Neuroblastoma Model after Treatment with Hypoxia-Mimetic Cobalt Chloride

**DOI:** 10.3389/fmicb.2018.00287

**Published:** 2018-02-21

**Authors:** Christine Brütting, Harini Narasimhan, Frank Hoffmann, Malte E. Kornhuber, Martin S. Staege, Alexander Emmer

**Affiliations:** ^1^Department of Surgical and Conservative Paediatrics and Adolescent Medicine, Martin Luther University of Halle-Wittenberg, Halle, Germany; ^2^Department of Neurology, Martin Luther University of Halle-Wittenberg, Halle, Germany; ^3^Department of Neurology, Hospital “Martha-Maria” Halle-Dölau, Halle, Germany

**Keywords:** endogenous retroviruses, open reading frames, *ERVFRD-1*, HERV-FRD, human endogenous retrovirus group FRD member 1, hypoxia, multiple sclerosis, neural precursor cell expressed developmentally down-regulated protein 9 (*NEDD9*)

## Abstract

Human endogenous retroviruses (ERVs) have been found to be associated with different diseases, e.g., multiple sclerosis (MS). Most human ERVs integrated in our genome are not competent to replicate and these sequences are presumably silent. However, transcription of human ERVs can be reactivated, e.g., by hypoxia. Interestingly, MS has been linked to hypoxia since decades. As some patterns of demyelination are similar to white matter ischemia, hypoxic damage is discussed. Therefore, we are interested in the association between hypoxia and ERVs. As a model, we used human SH-SY5Y neuroblastoma cells after treatment with the hypoxia-mimetic cobalt chloride and analyzed differences in the gene expression profiles in comparison to untreated cells. The vicinity of up-regulated genes was scanned for endogenous retrovirus-derived sequences. Five genes were found to be strongly up-regulated in SH-SY5Y cells after treatment with cobalt chloride: clusterin, glutathione peroxidase 3, insulin-like growth factor 2, solute carrier family 7 member 11, and neural precursor cell expressed developmentally down-regulated protein 9. In the vicinity of these genes we identified large (>1,000 bp) open reading frames (ORFs). Most of these ORFs showed only low similarities to proteins from retro-transcribing viruses. However, we found very high similarity between retrovirus envelope sequences and a sequence in the vicinity of neural precursor cell expressed developmentally down-regulated protein 9. This sequence encodes the human endogenous retrovirus group FRD member 1, the encoded protein product is called syncytin 2. Transfection of syncytin 2 into the well-characterized Ewing sarcoma cell line A673 was not able to modulate the low immunostimulatory activity of this cell line. Future research is needed to determine whether the identified genes and the human endogenous retrovirus group FRD member 1 might play a role in the etiology of MS.

## Introduction

**FIGURE 1:**
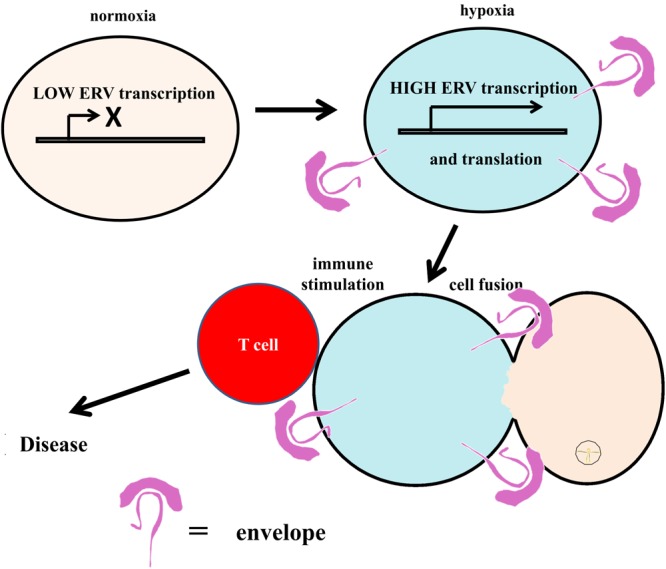
A working model for ERV reactivation. ERVs constitute an integral part of our genome. Under normal conditions, expression of ERVs is switched off epigenetically. Triggered by diverse factors like hypoxia, reactivation of ERV expression can be induced. ERV-encoded proteins can act as immunostimulatory superantigens or induce cytopathic effects, e.g., cell fusion.

Endogenous retroviruses (ERVs) are viral elements that are present in the genomes of virtually all species including human beings (Hayward and Katzourakis, 2015). At least 8% of the human genome is composed of endogenous retroviral sequences (Lander et al., 2001). These sequences were integrated into the human genome in the course of the evolution (Emerman and Malik, 2010). The great majority of ERVs are stabilized in the genome, but there is still ongoing or potential ERV genotype modification from parents to offspring through generations. Like other genes, ERVs are susceptible to mutations and proviral DNAs are predisposed to accumulate mutations as these sequences are usually not vital for the host survival and thus not under strong selective pressure. The majority of ERVs integrated in our genome is not competent to replicate and most ERV sequences are presumably silent (Jern and Coffin, 2008). Nevertheless, about one third of all ERV sequences in the genome were found to be transcriptionally active (Pérot et al., 2012). Some of these sequences still have open reading frames (ORFs) and, therefore, have the potential to code for a protein or peptide (Dupressoir et al., 2012; Wildschutte et al., 2016). ERVs can be reactivated by some herpes viruses such as Epstein–Barr virus (Mameli et al., 2012). Another possibility is the reactivation of ERV expression by hypoxia (Kewitz and Staege, 2013; Kulkarni et al., 2017). ERV-encoded superantigens might lead to hyper-stimulation of the immune system and tissue damage. In addition, fusogenic activity of ERV envelope proteins might have direct cytopathic effects which might be involved in MS pathogenesis independent on autoimmune mechanisms. Indeed, cell fusion has been detected in MS brain lesions as well as in animal models of MS (Kemp et al., 2012; Sankavaram et al., 2015). A working model for ERV reactivation and consequences is presented in **Figure 1**.

Endogenous retroviruses have contributed to certain physiological genes (i.e., syncytins) through modifications (Blond et al., 1999; Mi et al., 2000; Soygur and Moore, 2016) and can sometimes probably protect the host against exogenous retrovirus infections (Malfavon-Borja and Feschotte, 2015). On the other hand, ERVs have also been found to be associated with different diseases (Dolei, 2006; Balada et al., 2009), e.g., schizophrenia and bipolar disorder (Perron et al., 2012), type 1 diabetes mellitus (Mason et al., 2014), or cancer (Goering et al., 2015) as well as multiple sclerosis (MS) (Perron and Lang, 2010; De la Hera et al., 2014). Several ERVs are considered to be associated with MS (Christensen, 2010). For example, human ERV-W envelope mRNA expression was found to be selectively up-regulated in brain tissue from individuals with MS as compared with controls (Antony et al., 2004). In addition, HERV-H Env and HERV-W Env are increased on the surface of B cells and monocytes of MS patients (Brudek et al., 2009).

Multiple sclerosis is a chronic immune-mediated inflammatory disease of the central nervous system with characteristic patchy demyelination. It is the most common chronic disabling CNS disease in young adults and affects about 2.3 million people around the world (Browne et al., 2014). The etiology of MS has not been completely decoded so far; the causes of MS are hypothesized to be multifactorial including environmental influences (Islam et al., 2007) as well as epigenetic and genetic factors (Küçükali et al., 2015; Booth and Parnell, 2017). Commonly an autoimmune attack against myelin autoantigens is considered as the main occurrence in the pathogenesis of MS (Hemmer et al., 2003; Pender and Greer, 2007). Additionally, ERVs are discussed to contribute to MS (Tselis, 2011; Emmer et al., 2014). Besides, MS has been linked to hypoxia for decades (e.g., Fischer et al., 1983; Auer et al., 1995; Trapp and Stys, 2009). Hypoxic damage is hypothesized to be a factor in MS pathogenesis, because some patterns of demyelination are similar to white matter ischemia (Lassmann, 2003).

In the present study, we analyzed the effect of hypoxia-mimetic cobalt chloride (CoCl_2_) on human neuronal-like SH-SY5Y neuroblastoma cells for changes in gene expression profiles in contrast to un-stimulated cells. Genes up-regulated in this model are considered to indicate transcriptionally active chromatin regions which are susceptible also for ERV reactivation. Therefore, the vicinity of up-regulated genes was scanned for endogenous retrovirus sequences in order to identify possible ERV that might be involved in the link between hypoxia and MS. In addition, we analyzed the possible immune modulatory activity of the identified syncytin 2 in the A673 cell line system. We used this system because the immunostimulatory activity of A673 cells is well-characterized (Staege et al., 2004; Max et al., 2014; Reuter et al., 2015) and they display similar gene expression and splicing features as neuronal cells (Bros et al., 2006). The immunostimulatory activity of this model cell line has been shown to be susceptible to transgenic expression of varying molecules like interleukin 2 (Staege et al., 2004), CD137 ligand (Max et al., 2014), or OX40 ligand (Reuter et al., 2015).

## Materials and Methods

### Cell Lines and Cell Culture

Human SH-SY5Y neuroblastoma cells (Biedler et al., 1973) were obtained from the Deutsche Sammlung von Mikroorganismen und Zellkulturen (DSMZ, Braunschweig, Germany). A673 Ewing sarcoma cells (Giard et al., 1973) were obtained from the American Type Culture Collection (Manassas, VA, United States) All cells were cultured in Dulbecco’s Modified Eagle Medium (DMEM, PAA, Pasching, Germany), supplemented with 10% fetal calf serum, 100 U/mL penicillin, and 100 μg/mL streptomycin at 37°C in a humidified atmosphere with 5% CO_2_. For simulation of hypoxia, a fresh stock solution (10 mM) of CoCl_2_ was prepared in water and added to the medium to obtain desired final concentrations. SH-SY5Y cells were treated for 24 h at a cell density of 1 × 10^6^ cells/mL with either 0 μM CoCl_2_, 100 μM CoCl_2_, or 200 μM CoCl_2_. The experiment was repeated twice for the gene expression analysis with microarrays and three times for the gene expression analysis with polymerase chain reaction (PCR).

### Gene Expression Analysis

RNA was isolated using GeneMatrix Universal RNA Kit (roboklon, Berlin, Germany). RNA extracted from the cells was treated with DNase (roboklon, Berlin, Germany) to remove genomic DNA. Occasionally absence of DNA contamination was proved by using isolated RNA without reverse transcription as template for PCR. Global gene expression in SH-SY5Y cells was analyzed using Affymetrix Human Exon 1.0ST arrays (Affymetrix, Santa Clara, CA, United States). Affymetrix cel files were processed with Expression Console 1.1 (Affymetrix) at gene level (core; library version: huex-1_0-st-v2.na36.1.hg19). Calculations were performed with the MAfilter software (Winkler et al., 2012). Values of cobalt (II) chloride treated samples had to be three times higher than controls and signal intensities (RMA normalized, linear values) had to be above 100 to be considered as differentially expressed. Analysis was performed separately for cells treated with 100 μM CoCl_2_, or 200 μM CoCl_2_. For further analysis we included all threefold up-regulated genes that were found in both replications. Microarray cell files have been submitted to the Gene Expression Omnibus (GEO) data base (GSE107333).

### Identification of Endogenous Retrovirus Sequences

The chromosomal locations of the up-regulated genes were analyzed for the presence of putative ERV sequences essentially as described (Brütting et al., 2016). For this end, we analyzed the 2 Mbp surrounding each individual gene for the presence of ORFs with a minimal length of 1 kb by using Mobyle 1.5 (Rice et al., 2000). Identified ORFs were analyzed using BLASTP (Altschul et al., 2005) against the NCBI database of retro-transcribing viruses (taxid 35268) with the reference genome GRCh38 (primary assembly).

### Polymerase Chain Reaction

One microgram of the isolated RNA was transcribed into cDNA and used as a template for PCR. Real-time quantitative reverse transcription-PCR (qRT-PCR) was performed using Go Taq pPCR master mix (Promega, Mannheim, Germany) using 10 μL Go Taq pPCR master mix, 7 μL water, 1 μL forward primer, 1 μL reverse primer (25 μM) and 1 μL cDNA. PCR conditions were: 94°C, 30 s; 60°C, 30 s; 72°C, 45 s (40 cycles). Gene expression was calculated with the 2^-ΔΔCt^ method (Livak and Schmittgen, 2001). Conventional PCR was performed using 2 μl of the cDNA, 5 μl Green GoTaq Buffer (Promega, Mannheim, Germany), 0.5 μl of 10 mM dNTPs (Fermentas, Sankt Leon-Rot, Germany), 0.25 μl of each of the two primers (25 μM), 0.2 μl GoTaq polymerase (5 U/μl; Promega) and 16.8 μl water. All used primer sequences are listed in **Table 1**. The amplification protocol included an initial denaturation step at 95°C for 5 min, followed by 40 cycles with denaturation at 95°C for 60 s; primer annealing at 60°C for 60 s; amplification at 72°C for 90 s; and a final extension step at 72°C for 5 min. PCR products were subjected to agarose gel (1.5%) electrophoresis in the presence of ethidium bromide.

**Table 1:** Primer combinations used in this study.

Target^a^	Primer sequences (5′–3′)
ACTB	TAC AAT GAG CTG CGT GTG GC
	CGG ACT CGT CAT ACT CCT GC
HERV-FRD	CCC TCA CCC CCT TAT TTC AT
	TTT GAA GGA CTA CGG CTG CT
HERV-FRD^b^	ACC ATG GGC CTG CTC CT
	TCC TCC TTA GAA GGG TGA CTC
EWSR1-FLI1	GGC CAA GAT CAA TCC TCC AT
	ATG GAG GAT TGA TCT TGG CC
LIPI	AAC CAG CCC AAT CAG ACA AC
	AAT CAC TGG CCA GGA CAT TC
NEDD9	CAC CGC AGT GCT TAA TGC TG
	TCA CGG GGG TTA TCA CCT TTT T

*If not otherwise stated, primer combinations were used for qRT-PCR.*

*^*a*^ACTB, actin beta; HERV-FRD, syncytin 1; EWSR1-FLI1, Ewing sarcoma breakpoint region 1-Friend leukemia virus integration site 1; tumor specific gene fusion; LIPI, lipase member I (cancer-testis antigen 17); NEDD9, neural precursor cell expressed developmentally down-regulated protein 9.*

*^*b*^Used for conventional PCR only.*

### Cloning of *HERV-FRD* in pIRES2-AcGFP1 Vector

DNA (PCR product from SH-SY5Y cells) from the agarose gel was extracted with GeneJet Gel Extraction Kit (Thermo Fisher, Waltham, MA, United States), ligated in vector pGEM-T Easy (Promega) and transformed in *Escherichia coli* XL1-Blue. The DNA of one overnight colony was isolated with GeneJET Plasmid Miniprep Kit (Thermo Fisher, Waltham, MA, United States). DNA and vector pIRES2-AcGFP1 (Clontech, Mountain View, CA, United States) were digested with *Sac*I and *Sac*II. After agarose gel purification, ligation, and transformation into *Escherichia coli* XL1-Blue, individual clones were sequenced by using HERV-FRD specific primers. For sequencing, a 10 μL sequencing mix was used that contained 6.8 μL HPLC water, 0.2 μL sequence-specific sequencing primers (10 μM), 2.0 μL BigDyeTerminator v1.1 Cycle Sequencing buffer (Applied Biosystems, Foster City, CA, United States), 2.0 μL BigDyeTerminator v1.1 Cycle Sequencing Mix and 10 ng DNA. Sequence analysis was performed using ABI Prism^TM^ 310 Genetic Analyzer (Applied Biosystems). A clone with complete HERV-FRD ORF was used for further analysis. This clone differs from the reference sequence by a silent C to T transition (corresponding to base 1,384 in reference sequence NM207582).

### Transfection

For transient expression, SH-SY5Y cells and A673 cells were cultured for 24 h and then transfected with the appropriate vectors using PromoFectin (PromoKine, Heidelberg, Germany) according to the manufacturer’s protocol. For stable expression, cells were treated in the same way. After 24 h they were put under selection with the antibiotic G418.

### Mixed Lymphocyte Tumor Cell Culture (MLTC) and Flow Cytometry

Peripheral blood mononuclear cells (PBMC) were prepared and mixed lymphocyte tumor cell culture (MLTC) was performed as described elsewhere (Staege et al., 2004; Foell et al., 2008). Detection of surface antigens on PBMC by flow cytometry was performed as described elsewhere (Hoennscheidt et al., 2009). The following phycoerythrin labeled antibodies have been used: anti-CD3 clone SK7, anti-CD8 clone RPA-T8, and anti-CD25 clone 2A3. All antibodies were purchased from Becton Dickinson (Heidelberg, Germany) and all samples were analyzed on a FACScan instrument (Becton Dickinson) using CellQuestPro software (Becton Dickinson).

## Results and Discussion

According to our stringent filter criteria (see section “Materials and Methods”), only five genes were found to be strongly up-regulated in SH-SY5Y cells after treatment with cobalt chloride. These genes include (in alphabetical order) *CLU* (clusterin), *GPX3* (glutathione peroxidase 3), *IGF2* (insulin-like growth factor 2), *NEDD9* (neural precursor cell expressed, developmentally down-regulated 9), and *SLC7A11* [solute carrier family 7 (anionic amino acid transporter light chain, Xc-system), Member 11]. The up-regulated genes indicate transcriptionally active chromatin regions which might be susceptible for reactivation of other genetic elements like ERVs.

*CLU* (also known as apolipoprotein J, testosterone-repressed prostate message-2, or sulfated glycoprotein-2) encodes a glycoprotein which is nearly ubiquitously distributed in human tissues (Jones and Jomary, 2002). It is a 75–80 kDa heterodimer and a molecular chaperone which is normally secreted but in conditions of cellular stress, it can be transported to the cytoplasm where it can bind to BAX and inhibit neuronal apoptosis (Nuutinen et al., 2009). *CLU* expression has been associated with tumorigenesis of various malignancies, including tumors of the prostate, colon, and breast (Shannan et al., 2006). Variants in the clusterin gene are also associated with the risk of Alzheimer’s disease (Schrijvers et al., 2011), dementia (Weinstein et al., 2016), and stroke (Guido et al., 2015). In astrocytes of MS white matter lesions an elevated expression of clusterin was detected (van Luijn et al., 2015). All of these diseases represent states of increased oxidative stress, which in turn, promotes amorphous aggregation of target proteins, increased genomic instability and high rates of cellular death (Trougakos and Gonos, 2006).

*GPX3* (also known as plasma or extracellular glutathione peroxidase) encodes a protein which functions in the detoxification of hydrogen peroxide. Most of the *GPX3* mRNA is kidney-derived (Avissar et al., 1994), but it is also expressed by heart, lung, liver, brain, breast, and gastrointestinal tract (Chu et al., 1992; Tham et al., 1998). In human cancer *GPX3* promotor down-regulation and hyper-methylation is rather common (Zhang et al., 2010; Chen et al., 2011). *GPX3* expression and *GPX3* hyper-methylation can thus be used as biomarkers for different kind of cancer (Yang et al., 2013; Zhou et al., 2015). *GPX3* works as a tumor suppressor for example in colitis-associated carcinoma (Barrett et al., 2013) and in hepatocellular carcinoma (Qi et al., 2014). In initial MS lesions *GPX3* was found to be downregulated (>2 log_2_-fold) compared to control (Fischer et al., 2012).

*IGF2* encodes a protein with high homology to pro-insulin (Livingstone, 2013). IGF2 contains 10 exons and 4 promoters so that several alternatively spliced transcripts are possible (Engström et al., 1998). The *IGF2* gene is imprinted: the paternal *IGF2* allele is transcribed whereas the maternal allele is silent (Giannoukakis et al., 1993). As a growth factor it is especially expressed in many tissues in early stages of embryonic and fetal development (Hedborg et al., 1994). In adults, *IGF2* is preferentially expressed in liver and brain (Engström et al., 1998). IGF2 regulates normal cell growth and proliferation. Moreover, it plays a role in the growth and development of tumors: epigenetic changes at this locus are for example associated with Wilms tumor, Beckwith–Wiedemann syndrome, or rhabdomyosarcoma (Bergman et al., 2013).

*SLC7A11* (also known as xCT) encodes a protein that is member (together with *SLC3A2*) of a heterodimeric, sodium-independent, anionic amino acid transport system that is highly specific for cysteine and glutamate (Sato et al., 2000). While SLC7A11 seems to induce the transport activity, SLC3A2 leads to the surface expression of the system (Verrey et al., 2004). SLC7A11 seems to contribute to different kinds of cancer, including, e.g., malignant glioma (Robert et al., 2015) or breast cancer (Liu et al., 2011). In tumor cells, the amino acid transport system plays a critical role in regulating intracellular glutathione levels (Okuno et al., 2003) and glutathione has been broadly implicated in chemotherapy resistance (Gatti and Zunino, 2005). Besides, *SLC7A11* is significantly up-regulated in post-mortem spinal cord samples from MS patients (Lieury et al., 2014). SLC1A11 is a member of the solute carrier family, a large gene family that contains several receptors for retroviruses. Interestingly, two members of this family (SLC1A4, SLC1A5) have also been suggested as receptors for ERV (Lavillette et al., 2002). A function as receptor for viruses has not been described for SLC1A11.

*NEDD9* (also known as *CasL* and *HEF1*) encodes a protein which regulates diverse cellular processes that are relevant to cancer, like cell attachment, migration, invasion, apoptosis, or cell cycle regulation (Singh et al., 2007; Shagisultanova et al., 2015). Furthermore, *NEDD9* seems to play a role in the nervous system as there is some association between one *NEDD9* variation and the susceptibility of late-onset Alzheimer’s disease and Parkinson’s disease (Li et al., 2008). As it is involved in TGFβ-mediated differentiation into the neuronal lineage and *NEDD9* possibly promotes a progenitor status that renders the cells competent to differentiation into neurons (Vogel et al., 2010). It is enriched in neural progenitor cells (Abramova et al., 2005) and its down-regulation is linked to neuronal lineage commitment (Aquino et al., 2008).

Based on our search strategy (see section “Materials and Methods”), we found in the vicinity of the up-regulated genes large (>1,000 bp) ORFs (from 11 in the vicinity of *NEDD9* to 169 in the vicinity of *IGF2*). For all genes, these ORFs included candidates that passed the default threshold of the NCBI BLASTP implementation [expect (E) value < 10] against the database of retro-transcribing viruses. For four of the genes (all with the exception of SLC7A11) these BLASTP hits include envelope sequences from retro-transcribing viruses. The E-values for nearly all of these hits were higher than 0.01 and, therefore, are not convincing retroviral (ERV) sequences. However, we found one hit with very high similarity to retroviral envelope proteins in the vicinity of NEDD9 (see Supplementary Figure 1).

**FIGURE 2:**
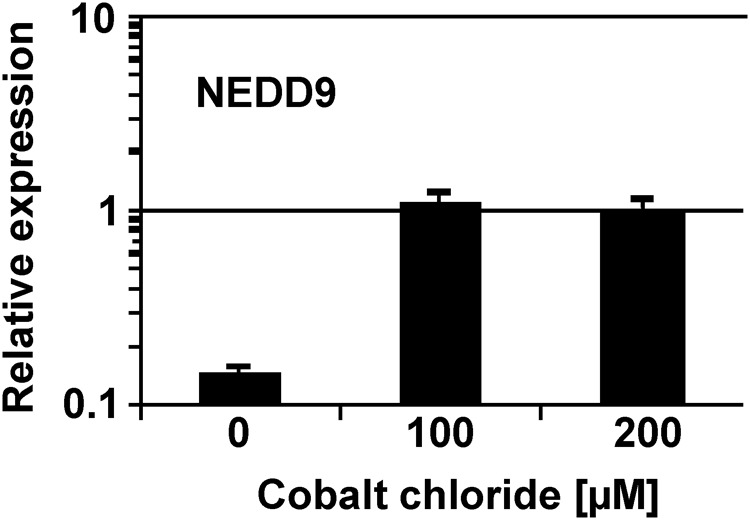
Expression of *NEDD9* in SH-SY5Y cells. Expression of *NEDD9* was analyzed in SH-SY5Y cells under different culture conditions by qRT-PCR. Cells were cultured in absence of CoCl_2_ (water control), with 100 μM of CoCl_2_ or with 200 μM of CoCl_2_. Presented are means and standard deviations from three independent experiments. For comparative analysis, beta actin was used as housekeeping control and the median expression of all samples was set as one.

We validated up-regulation of *NEDD9* in CoCl_2_ treated SH-SY5Y cells by qRT-PCR (**Figure 2**). Our results are in agreement with observations from other groups also demonstrating that *NEDD9* is induced by hypoxia (Martin-Rendon et al., 2007; Kim et al., 2010).

The BLASTP hit in the vicinity of *NEDD9* (accession number CAB94192.1; see Supplementary Figure 1) represents a sequence (“HERV-H/env62”) of the human HERV-H family. With about 1,000 elements the HERV-H family is one of the largest HERV families in the human genome (Wilkinson et al., 1994). Analyzes showed that there are three envelopes with large ORFs corresponding to potential 59-, 60-, and 62-kDa translational products (de Parseval et al., 2001). Moreover, the higher HERV seroreactivity in patients with active MS correlates with the higher levels of HERV-H Env expression on B cells and monocytes (Brudek et al., 2009).

**FIGURE 3:**
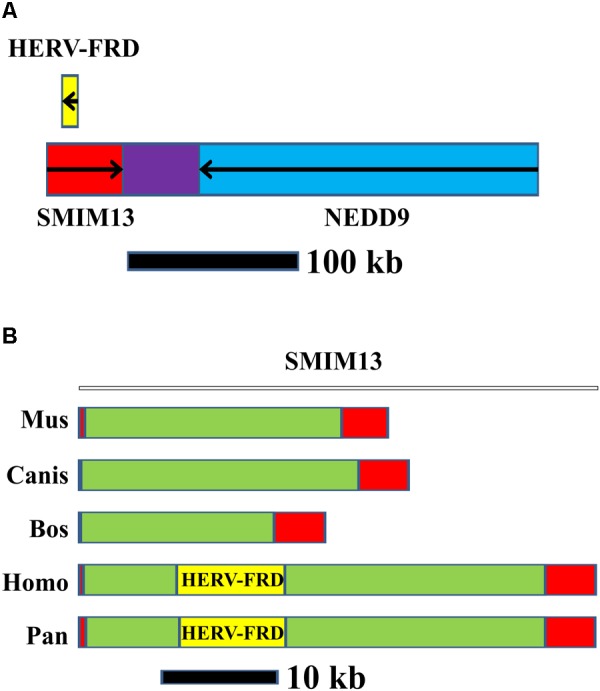
Organization of the *NEDD9/SMIM13/HERV-FRD* region in humans and other species. **(A)** *HERV-FRD* is located in the intron of *SMIM13*. SMIM13 and NEDD9 are located tail-to-tail orientated on human chromosome 6. **(B)** Comparison of the *SMIM13/HERV-FRD* region in different vertebrate species. Red: exons of *SMIM13;* green: *SMIM13* intron; yellow: *HERV-FRD.*

The sequence in the vicinity of *NEDD9* is identical to the human endogenous retrovirus group FRD, member 1 (*HERV-FRD*). *HERV-FRD* is located in an intron of the small integral membrane protein 13 (*SMIM13*). The close association between NEDD9 and SMIM13 is highly conserved in vertebrates. However, in non-primate vertebrates, *HERV-FRD* is absent (**Figure 3**). *HERV-FRD* entered the primate genomes more than 40 million years ago (de Parseval and Heidmann, 2005). It has inactivating mutations in the *gag* and *pol* genes whereas the envelope glycoprotein gene is preserved (Renard et al., 2005). The encoded protein product is called syncytin 2 (Blaise et al., 2003) which plays a major role in placental development and trophoblast fusion (Malassiné et al., 2007; Vargas et al., 2009). The protein has the characteristics of a typical retroviral envelope protein, including a cleavage site that separates the surface and transmembrane units which together form a heterodimer of the mature syncytin 2 (Renard et al., 2005). Syncytin 2 can induce cell-cell fusion (Blaise et al., 2003).

**FIGURE 4:**
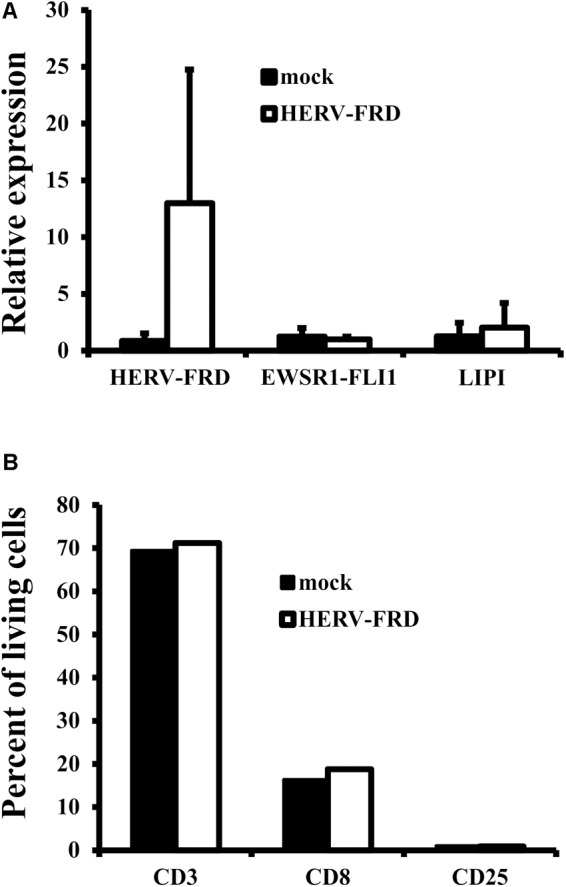
Absence of immunomodulatory effects of *HERV-FRD* transfected A673 cells. A673 cells were transfected with *HERV-FRD* or with empty vector (mock). **(A)** RNA from transfected cells were analyzed for presence of *HERV-FRD* and the Ewing sarcoma specific transcripts *EWSR1-FLI1* and *LIPI*. **(B)** Transfected cells were used as stimulatory cells in mixed lymphocyte/tumor cell cultures. Stimulated cells were analyzed for presence of T cells (CD3), cytotoxic T cells (CD8), and activated lymphocytes (CD25). Presented are means and standard deviations from three independent experiments.

In our model we found up-regulation only for the mentioned five genes and not for the associated ERVs and we have no evidence that ERVs are functionally involved in up-regulation of the genes or *vice versa*. From our data we only found HERV-FRD to be a candidate for a possible association between hypoxia and ERVs in MS. Other factors (e.g., patient specific polymorphisms) might be necessary to induce expression of the ERVs and subsequent effects. Under such conditions, it seems possible that over-expression of syncytin 2 in the brain, e.g., as a consequence of local hypoxia, elicits an immunomodulating activity. Therefore, we tested whether syncytin 2 overexpression lead to altered immunostimulatory activity in the well-characterized A673 model system (Staege et al., 2004; Reuter et al., 2015). HERV-FRD transfected A673 cells retained the expression of tumor associated antigens (**Figure 4A**). However, we were not able to find altered immunostimulatory activity of transfected cells (**Figure 4B**) in this system. Further investigations are needed to analyze possible immunomodulatory properties.

Taking together, our study shows changes in gene expression profiles of hypoxia-mimetic CoCl_2_ treated human neuronal-like SH-SY5Y cells in contrast to untreated cells. Five genes were found to be strongly up-regulated: CLU, GPX3, IGF2, NEDD9, and SLC7A11. Three of them (CLU, GPX3, and SLC7A11) showed in the past some associations to MS. The identified ERV in the vicinity of NEDD9 might thus be involved in the association between hypoxia and MS.

## Author Contributions

CB: data collection, data analysis, and interpretation, generating figures, and drafting the article. HN: part of data collection. FH and MK: conception of the work. MS: conception of the work, generating figures, and critical revision of the article. AE: conception of the work and final approval of the version to be published.

## Conflict of Interest Statement

The authors declare that the research was conducted in the absence of any commercial or financial relationships that could be construed as a potential conflict of interest.
