# Supplementary material for: Investigation of Endogenous Retrovirus Sequences in the Neighborhood of Genes Up-regulated in a Neuroblastoma Model after Treatment with Hypoxia-Mimetic Cobalt Chloride
Source: Front Microbiol. 2018 Feb 21;9:287. doi: 10.3389/fmicb.2018.00287 (PMC5826361; doi:10.3389/fmicb.2018.00287)
Supplement: Supplementary file 1 [file Data_Sheet_1.DOCX]

**Supplementary Figure 1.** BLASTP result for the open reading frame in vicinity to *NEDD9* and the non-redundant data base with search limited to records that include taxid 35268 (best hit)

envelope protein [HERV-H/env62]

Sequence ID: CAB94192.1Length: 584Number of Matches: 1

Alignment statistics for match #1

Score Expect Method Identities Positives Gaps

269 bits(688) 2e-84 Compositional matrix adjust. 196/542(36%) 289/542(53%) 58/542(10%)

Query 16 LAAYRHPDFPLLEKAQQLLQSTGSPYSTNCWLCTSSSTETPGTAYPASPREWTSIEAELH 75

L +Y H L LL ++ NCWLC S S+ TA PA +W + LH

Sbjct 38 LPSYLHHTINL---THSLLAASNPSLVNNCWLCISLSSSA-YTAVPAVQTDWATSPISLH 93

Query 76 I---------------------SYRWDPNLKGLMRPANSLLSTVKQDF-PDIRQKPPIFG 113

+ S + P++ + A +LL T ++ P I PPIFG

Sbjct 94 LRTSFNSPHLYPPEELIYFLDRSSKTSPDISH--QQAAALLRTYLKNLSPYINSTPPIFG 151

Query 114 PIFTNINLMGIAPICVMAKRKNGTNVGTLPSTVCNVTFTVDSNQQTYQTYTHNQFRHQPR 173

P+ T + AP+C+ +R G +G L + C+ T + S T T F Q

Sbjct 152 PLTTQTTIPVAAPLCISWQRPTGIPLGNLSPSRCSFTLHLRS-PTTNINETIGAF--QLH 208

Query 174 FPKPPNITFPQGTLLDKSSRFCQGRPSSCSTRNFWFRPADYNQ-------CLQISNLSST 226

P+I L + SS +C GR C + + W + CL I + +

Sbjct 209 ITDKPSIN--TDKLKNISSNYCLGRHLPCISLHPWLSSPCSSDSPPRPSSCLLIPSPENN 266

Query 227 AEWVLLDQTRNSLFWENKTKGANQ--SQTPCVQVLAGMTIATSYLGISAVSEFFGTSLTP 284

+E +L+D R + EN+T + Q Q+P +Q L +A S LG+ F T +

Sbjct 267 SERLLVDTRRFLIHHENRTFPSTQLPHQSP-LQPLTAAALAGS-LGVWVQDTPFSTP-SH 323

Query 285 LFHFHISTCLKTQGAFYICGQSIHQCLPSNWTGTCTIGYVTPDIFIAPGNLSLPIPIYGN 344

LF H+ CL QG F++CG S + CLP+NWTGTCT+ ++TP I A G LP+P+

Sbjct 324 LFTLHLQFCLA-QGLFFLCGSSTYMCLPANWTGTCTLVFLTPKIQFANGTEELPVPLMTP 382

Query 345 SPLPRVRRAIHFIPLLAGLGILAGT---GTGIAGITKASLTYSQLSKEIANNIDTMAKAL 401

+ + +R I IPL+ GLG+ A T GTGIAGI+ + +T+ LS + + +I +++ L

Sbjct 383 T---QQKRVIPLIPLMVGLGLSASTVALGTGIAGISTSVMTFRSLSNDFSASITDISQTL 439

Query 402 TTMQEQIDSLAAVVLQNRRGLDMLTAAQGGICLALDEKCCFWVNQSGKVQDNIRQLLNQA 461

+ +Q Q+DSLAAVVLQNRRGLD+LTA +GG+C+ L+E+CCF++NQSG V DNI++L ++A

Sbjct 440 SVLQAQVDSLAAVVLQNRRGLDLLTAEKGGLCIFLNEECCFYLNQSGLVYDNIKKLKDRA 499

Query 462 SSLRERATQGWLNWEGTW---KWFSWVLPLTGPLVSLLLLLLFGPCLLNLITQFVSSRLQ 518

L +A+ E W W SWVLP+ PL+ + LLLLFGPC+ L++QF+ +R+Q

Sbjct 500 QKLANQASN---YAEPPWALSNWMSWVLPIVSPLIPIFLLLLFGPCIFRLVSQFIQNRIQ 556

Query 519 AI 520

AI

Sbjct 557 AI 558

**Complete analyzed sequence (Query):**

VTMGLLLLVLILTPSLAAYRHPDFPLLEKAQQLLQSTGSPYSTNCWLCTSSSTETPGTAYPASPREWTSIEAELHISYRWDPNLKGLMRPANSLLSTVKQDFPDIRQKPPIFGPIFTNINLMGIAPICVMAKRKNGTNVGTLPSTVCNVTFTVDSNQQTYQTYTHNQFRHQPRFPKPPNITFPQGTLLDKSSRFCQGRPSSCSTRNFWFRPADYNQCLQISNLSSTAEWVLLDQTRNSLFWENKTKGANQSQTPCVQVLAGMTIATSYLGISAVSEFFGTSLTPLFHFHISTCLKTQGAFYICGQSIHQCLPSNWTGTCTIGYVTPDIFIAPGNLSLPIPIYGNSPLPRVRRAIHFIPLLAGLGILAGTGTGIAGITKASLTYSQLSKEIANNIDTMAKALTTMQEQIDSLAAVVLQNRRGLDMLTAAQGGICLALDEKCCFWVNQSGKVQDNIRQLLNQASSLRERATQGWLNWEGTWKWFSWVLPLTGPLVSLLLLLLFGPCLLNLITQFVSSRLQAIKLQTNLSAGRHPRNIQESPF
